# The impact of the mesoprefrontal dopaminergic system on the maturation of interneurons in the murine prefrontal cortex

**DOI:** 10.3389/fnins.2024.1403402

**Published:** 2024-07-05

**Authors:** K. Ushna S. Islam, Sandra Blaess

**Affiliations:** Neurodevelopmental Genetics, Institute of Reconstructive Neurobiology, Medical Faculty, University of Bonn, Bonn, Germany

**Keywords:** development, neuropsychiatric disease, monoaminergic innervation, parvalbumin, calbindin

## Abstract

The prefrontal cortex (PFC) undergoes a protracted maturation process. This is true both for local interneurons and for innervation from midbrain dopaminergic (mDA) neurons. In the striatum, dopaminergic (DA) neurotransmission is required for the maturation of medium spiny neurons during a critical developmental period. To investigate whether DA innervation influences the maturation of interneurons in the PFC, we used a conditional knockout (cKO) mouse model in which innervation from mDA neurons to the mPFC (mesoprefrontal innnervation) is not established during development. In this mouse model, the maturation of parvalbumin (PV) and calbindin (CB) interneuron populations in the PFC is dysregulated during a critical period in adolescence with changes persisting into adulthood. PV interneurons are particularly vulnerable to lack of mesoprefrontal input, showing an inability to maintain adequate PV expression with a concomitant decrease in *Gad1* expression levels. Interestingly, lack of mesoprefrontal innervation does not appear to induce compensatory changes such as upregulation of DA receptor expression in PFC neurons or increased innervation density of other neuromodulatory (serotonergic and noradrenergic) innervation. In conclusion, our study shows that adolescence is a sensitive period during which mesoprefrontal input plays a critical role in promoting the maturation of specific interneuron subgroups. The results of this study will help to understand how a dysregulated mesoprefrontal DA system contributes to the pathophysiology of neurodevelopmental disorders.

## Introduction

The medial prefrontal cortex (mPFC) is involved in higher-order cognitive functions such as planning, inhibitory control, decision-making, and working memory. The executive functions of the mPFC are governed by its primary function of integrating information to execute actions according to internally represented goals, and depend on its extensive interactions with other brain regions (Fuster, 2001; Miller et al., 2002). Modulatory innervation to the mPFC, including cholinergic, noradrenergic, serotonergic and dopaminergic inputs from subcortical structures contribute to sustaining mPFC functions. The dopaminergic (DA) input to the mPFC originates from a subset of midbrain dopaminergic (mDA) neurons in the ventral tegmental area (VTA) (Islam et al., 2021; Reynolds and Flores, 2021). This so-called mesoprefrontal DA system is essential to maintain the working memory function of the mature mPFC (Cools and Arnsten, 2022).

Compared to the sensory and motor cortex, the mPFC, and especially its GABAergic interneurons, are subject to a protracted process of functional and anatomic maturation, which lasts until early adulthood. This maturation ultimately leads to the correct calibration of the excitation/inhibition (E/I) balance in mPFC neuronal networks and the acquisition of mature cognitive functions (Caballero and Tseng, 2016). Changes in the developmental trajectories of GABAergic interneurons may contribute to susceptibility to psychiatric disorders such as schizophrenia, which usually first emerge during adolescence and lead to deficits in the domain of executive functions mediated by the mPFC (Schubert et al., 2015; Caballero and Tseng, 2016; Chini and Hanganu-Opatz, 2020).

GABAergic interneurons can be classified into subpopulations with distinct morphological, molecular, and functional properties (Rudy et al., 2011; Lim et al., 2018). Interneurons expressing the calcium-binding proteins calretinin (CR), calbindin (CB), or parvalbumin (PV) constitute 80% of the GABAergic interneuron population in the rodent mPFC (Gabbott et al., 1997). Protein levels and the number of interneurons expressing these markers stabilize between adolescence and young adulthood: CR expression decreases, while PV and CB expression increases (Caballero et al., 2014a; Caballero and Tseng, 2016; Du et al., 2018). This is accompanied by functional changes: for example, PV-expressing fast-spiking interneurons experience doubling of incoming excitatory post-synaptic currents frequency during this critical period, accompanied by a surge in inhibitory post-synaptic potentials onto the pyramidal neurons of layers V-VI (Caballero and Tseng, 2016; Ferguson and Gao, 2018; Larsen and Luna, 2018). The mechanisms orchestrating the maturation processes of mPFC interneurons are not fully understood.

Similar to overall mPFC development, the development of the mesoprefrontal DA system is also relatively protracted. DA fibers in the mPFC are sparse at prenatal stages and innervation density increases slowly during the postnatal phase to eventually stabilize with the onset of adulthood (Islam et al., 2021; Reynolds and Flores, 2021). In the striatum, DA neurotransmission during a specific postnatal time window promotes spiny medium neuron maturation (Lieberman et al., 2018) and stimulating DA signaling in the mPFC of schizophrenia mouse models during a critical postnatal period ameliorates altered interneuron phenotypes and circuit function (Mukherjee et al., 2019; Mastwal et al., 2023). Thus, the proper establishment of the mesoprefrontal DA input during development may play a key role in the maturation of local mPFC microcircuits.

To investigate whether the mesoprefrontal DA pathway influences the maturation of GABAergic interneurons in the mPFC, we used a conditional knockout (cKO) mouse model in which the mesoprefrontal DA projections are not established during development and mPFC dopamine levels are severely reduced (Kabanova et al., 2015). In this mouse model, we investigated the maturation profile of PV, CB and CR interneurons based on their marker expression, and possible compensatory changes such as upregulation of dopamine receptor expression or an increase in serotonergic and noradrenergic innervation of the mPFC.

## Methods

### Mouse lines

Mice were housed in a controlled environment with 12 hr light/night cycles and access to food and water *ad libitum*. Mouse lines were maintained on a mixed CD1/C57BL6 background. *En1*^*Cre*/+^; *Gli2*^*zfd*/+^ mice were crossed with *Gli2*^*flox*/+^ mice to generate *En1*^*Cre*/+^; *Gli2^zfd/flox^* (*Gli2* cKO) or *En1*^*Cre*/+^; *Gli2*^*zfd*/+^ (control) mice (Kabanova et al., 2015). A cross between Gli2*^zfd^* and Gli2*^flox^* was chosen to generate the *Gli2* cKO mice, so that Cre recombinase only needs to recombine one allele of *Gli2*. Importantly, *En1* and *Gli2* are separated by only 1.1 cM on chromosome 1; thus, *En1^Cre^* and *Gli2^zfd^* are linked (Corrales et al., 2006). *En1^Cre^* mice are heterozygous for *En1* and *En1* heterozygosity has been reported to cause progressive degeneration of mDA neurons starting at 8 weeks of age (Sonnier et al., 2007). To exclude any confounding effects of the potential neurodegenerative phenotype of *En1^Cre^* mice on the parameters analyzed in our study, double heterozygous mice (*En1*^*Cre*/+^; *Gli2*^*zfd*/+^) were used as controls. Brain structures in *Gli2* heterozygous mice have been described as phenotypically indistinguishable from their wild-type littermates (Heyne et al., 2016). Experiments were performed in compliance with the regulations for the welfare of animals issued by the Federal Government of Germany, European Union legislation and the regulations of the University of Bonn. The protocols were approved by the LANUV NRW.

### Tissue processing, immunostaining and fluorescent RNA *in situ* hybridization (RNA-FISH)

Mice were anesthetized with an intraperitoneal injection of Ketanest/Rompun and perfused transcardially with phosphate buffered saline (PBS), followed by 4% PFA. Brains were cryosectioned at 40 μm and collected as free-floating sections in anti-freeze solution.

*Immunostaining*: Sections were incubated in blocking buffer (10% normal donkey serum (NDS) with 0.3% Triton in PBS (PBT)) for 1 h at room temperature (RT) followed by primary antibody in 3% NDS, 0.3% PBT overnight at 4°C. Sections were washed with 0.3% PBT and incubated with secondary antibody in 3% NDS, 0.3% PBT and Hoechst for 2 h at RT. For biotinylated secondary antibodies, sections were additionally incubated in fluorophore-conjugated streptavidin solution for 1h at RT. Antibodies and dilutions are provided in Supplementary Table 1.

*RNA-FISH*: Sections were mounted on slides, dried at 40°C for 30 min, post-fixed in 4% PFA, dehydrated in a graded series of ethanol and dried at 40°C for 20 min. FISH was performed using RNAscope Fluorescent Multiplex Detection Reagents (ACDBio) according to the manufacturer’s instructions for frozen tissue (User Manual: 323100-USM). Hybridized probes (Supplementary Table 1) were detected with TSA Cyanine 3 or 5 (Perkin Elmer). Cell nuclei were counterstained with DAPI. For combined *in situ* hybridization and immunofluorescence staining, sections were first immunostained and then subjected to the RNA-FISH protocol.

### Image acquisition

Images were acquired either as single plane with epifluorescence microscopes (Zeiss AxioObserver Z1, Zeiss Axio Scan.Z1) or as Z-stacks with confocal microscopes (Zeiss AxioObserver with CSU-W1 confocal scanner unit, Leica TCS SP8). Details are provided in Supplementary Table 2. Acquisition parameters were kept constant for controls and *Gli2*-cKO mice. Z-stacks are presented as maximum intensity projections (MIP).

### Image analysis

Image analysis was performed in mPFC at bregma level 1.78–1.54 mm, except for analysis of CB cell density (bregma level 2.1–1.98 mm). For all analyses, mPFC was traced by overlaying the corresponding bregma level from the mouse brain atlas (Paxinos and Franklin, 2000) and divided into upper and deeper layers using ImageJ (Schneider et al., 2012).

#### Cell density of CB, PV, CR interneurons

To reduce background and out-of-focus signal (in widefield images), the mean gray value (MGV) (CR, PV) or a fourth of the MGV (CB) was subtracted. Labeled cells were identified based on clearly recognizable cell morphology in the focal plane, manually counted using the ImageJ ‘Cell Counter’ plugin and normalized for the area. CB-positive cells are dense and clustered in the upper layers of the mPFC, thus our analysis might underestimate the number of CB positive cells in the upper layers. However, the same criteria for cell identification (see above) were applied for both control and cKO mice and across all time points analyzed. In addition, the experimenter was blinded to the genotype for most of the stages analyzed (see below).

#### PV, fluorescence intensity

The soma of the immunolabeled PV neurons was marked at the spot exhibiting optimal staining. Intensity values were recorded with the ImageJ ‘Cell Counter’ plugin. Intensity values obtained from corpus callosum within the same image were subtracted for background correction.

#### Monoaminergic fibers

Each acquisition area (175.9 μm × 175.9 μm) was processed using the Digital Enhancement of Fibers with Noise Elimination (DEFiNE) macro for Fiji (Powell et al., 2019). In short, stacked images were converted into a binary mask and large particles were removed. Z-stacks were converted to MIPs and smaller fluorescent artifacts were removed. Ten 12 × 12 μm regions without labeled fibers were manually selected to measure mean pixel intensity and standard deviations of the selected regions. A threshold of 4 standard deviations was set above the mean background pixel intensity and labeled axonal fibers with intensity above the defined threshold were recorded to compute the total area occupied by the fibers.

#### RNA-FISH signal

CellProfiler (Stirling et al., 2021) was used for a non-biased automated analysis of fluorescent signal. The pipelines used for analysis were based on ‘Speckle Counting’^1^ and adjusted and optimized for individual experiments (Supplementary Table 2).

#### Blinding

For quantification of interneuron cell density, experimenters were blinded to genotype for the following conditions: CR (P19, P33, P60) and CB (P12, P19, P26, P33, P60).

### Statistical analysis

Data are presented as mean ± standard error of the mean (s.e.m). Statistical analyses were performed with GraphPad Prism 9 and 10 (GraphPad). P values of less than 0.05 were considered statistically significant. Details on the number of analyzed animals or cells and statistical tests are reported in Supplementary Tables 3, 4.

## Results

### Mouse model lacking mesoprefrontal dopaminergic innervation

During embryogenesis, Sonic hedgehog (SHH) signaling is essential for the induction of mDA progenitors between embryonic day (E)8.0 and E10.5, in a temporal-spatial dynamic manner. Conditional inactivation of the gene encoding the transcription factor GLI2, which is a downstream effector in the SHH signaling pathway, around E9.0 in the midbrain largely inactivates SHH signaling activity in mDA progenitors (Blaess et al., 2006). To achieve this spatial and temporal conditional inactivation of *Gli2*, *En1*^*Cre*/+^; *Gli2*^*zfd*/+^ mice were crossed with *Gli2*^*flox*/+^ mice to generate *En1*^*Cre*/+^; *Gli2^zfd/flox^* (*Gli2* cKO) or *En1*^*Cre*/+^; *Gli2*^*zfd*/+^ (control) mice. In *Gli2*-cKO mice, mDA progenitors that give rise to mDA in the medial ventral tegmental area are severely reduced and mesoprefrontal DA projections fail to form. In contrast, mDA projections to other target areas such as the striatum, nucleus accumbens and amygdala are not significantly reduced (Kabanova et al., 2015).

### Interneuron maturation is altered in absence of mesoprefrontal dopaminergic innervation

**FIGURE 1:**
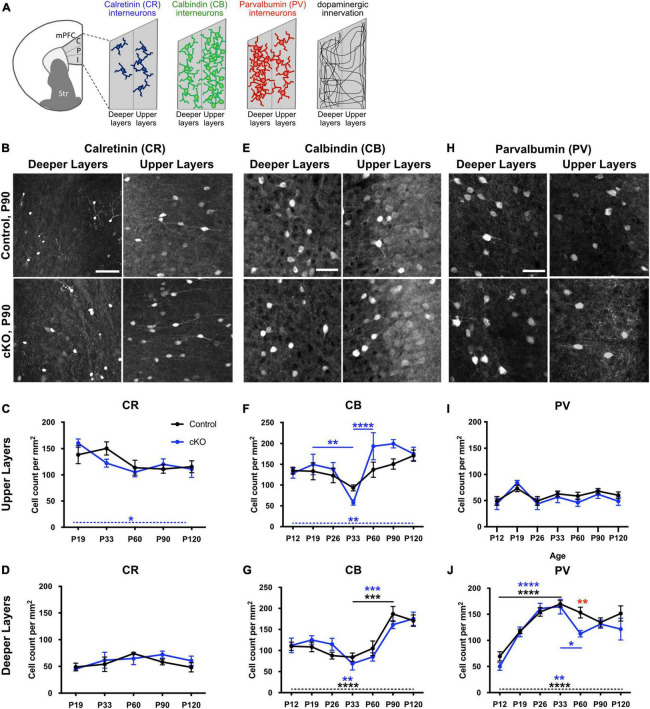
Development of interneuron density throughout postnatal development is altered in absence of mesoprefrontal dopaminergic innervation in the mPFC. **(A)** Diagram showing the mPFC and its subregions (C: cingulate, P: prelimbic, I: infralimbic) and the relative distribution of CR+, CB+ and PV+ interneurons and of TH+ fibers in upper and deeper layers. **(B–J)** Representative images of immunofluorescent staining for CR **(B)**, CB **(E)** and PV **(H)** in deeper and upper layers of the P90 prelimbic cortex (coronal sections; scale bars: 50 μm) and quantification of cell density for the different groups of interneurons in upper and deeper layers in the mPFC of control (black) and *Gli2*-cKO (blue) mice across postnatal development **(C,D,F,G,I,J)**. **(C,D)** CR+ interneurons. *n* = 3–5 mice per group. There is no significant change in cell density during development in upper or deeper mPFC layers in control mice, but a significant change over different developmental stages in the upper layer of *Gli2*-cKO (cKO) mice (**C**, dashed blue line and blue asterisk). Two-way ANOVA with age and genotype as the two main factors and one-way ANOVA followed by a post-hoc test for linear trend. **(F,G)** CB+ interneurons. *n* = 4–6 mice per group. **(F)** Upper layers: While there is no significant variation in cell density throughout postnatal development in control animals, cell density in *Gli2*-cKO animals shows a significant drop between P19 and P33 and a highly significant increase between P33 and P60 (blue asterisks and solid blue lines). Two-way ANOVA with age and genotype as the two main factors followed by Šídák’s multiple comparisons *post-hoc* test. One-way ANOVA followed by a *post-hoc* test for linear trend shows a significant increase in cell density from P12 to P120 in *Gli2*-cKO mice (dashed blue line and blue asterisk) but not in control mice. **(G)** Deeper layers: cell density in cKO and control mPFC is relatively constant between P12 and P33, increases significantly between P33 and P90 and plateaus at P90. Two-way ANOVA with age and genotype as the two main factors followed by Šídák’s multiple comparisons *post-hoc* test. One-way ANOVA followed by a *post-hoc* test for linear trend shows a significant increase in cell density from P12 to P120 in cKO and in control mice (dashed line, blue asterisks indicate significance in *Gli2*-cKO, black asterisks indicate significance in control). **(I,J)** PV+ interneurons. *n* = 5–8 mice per group. **(I)** Upper layers: There is no significant variation in cell density across postnatal development in control or *Gli2*-cKO mice. Two-way ANOVA with age and genotype as the two main factors followed by Šídák’s multiple comparisons *post-hoc* test and one-way ANOVA followed by a *post-hoc* test for linear trend across age. **(J)** Deeper layers: PV cell density increases significantly between P12 and P33 in cKO and control mPFC. In control animals, the cell density decreases slightly but not significantly between P33 and P90, while there is a significant drop between P33 and P60 in the *Gli2*-cKO group. Two-way ANOVA with age and genotype as the two main factors followed by Šídák’s multiple comparisons *post-hoc* test. Red asterisk indicates a significant change in the number of PV interneurons between the *Gli2*-cKO and control group at P60. One-way ANOVA followed by a *post-hoc* test for linear trend shows a significant increase in cell density from P12 to P120 in cKO and in control mice (dashed line, blue asterisks indicate significance in *Gli2*-cKO, black asterisks indicate significance in control). Details on animal numbers are reported in Supplementary Table 3; details on statistical tests in Supplementary Table 4. Error bars indicate mean +/- SEM. *p < 0.05, **p < 0.01, ***p < 0.001, ****p < 0.0001.

To investigate whether the absence of mesoprefrontal DA innervation affects the maturation of interneurons in the mPFC, we analyzed the density of cells expressing the calcium-binding proteins CR, CB and PV in the mPFC at multiple developmental stages, ranging from the early postnatal (P) phase (P12) to full adulthood (P120) in *Gli2*-cKO mice and controls. For this analysis, the mPFC was segmented into upper and deeper layers because of the distinct density of these interneuron types and of DA input in upper and deeper layers (Figure 1A; Weele et al., 2018).

Analysis of the density of CR-expressing cells in control and *Gli2*-cKO mPFC revealed no significant effect of genotype and no genotype x age interaction in either layer (two-way ANOVA). In deeper layers, CR-expressing cells are relatively sparse and neither control nor *Gli2*-cKO mice showed any significant changes across the analyzed age groups. In the upper layers, a significant effect of age was detected (two-way ANOVA, *F*_(4, 35)_ = 8.898, *p* = 0.0113) and one-way ANOVA followed by a test for linear trend showed a significant systematic decrease in CR cell density form P19 to P120 in *Gli2*-cKO but not in control animals (Figures 1B–D and Supplementary Tables 3, 4).

CB immunostaining revealed that CB-expressing cells are present throughout layers II-VI but are most prominent in layer II and III (Figures 1E–G and Supplementary Figure 1). Comparison of the density of CB-expressing cells in control and *Gli2*-cKO mPFC demonstrated no significant effect of genotype and no genotype x age interaction in either the upper or deeper layers (two-way ANOVA), but a significant effect of age in deeper (*F*_(6, 53)_ = 15.58, *p* < 0.0001) and upper (*F*_(6, 53)_ = 7.269, *p* < 0.0001) layers. In the deeper layers, the CB cell density remained relatively stable until P60 and then increased significantly both in control and in *Gli2*-cKO mice. In addition, one-way ANOVA followed by a test for linear trend showed a significant systematic increase in CB cell density from P12 to P120 in the deeper layers of *Gli2*-cKO and in control animals (Figure 1G and Supplementary Tables 3, 4). In the upper layers of control mice, the density of CB-expressing cells showed no significant changes during postnatal development. In contrast, cell density was found to vary considerably between P26 and P60 in *Gli2*-cKO mice: CB cell density dipped at P33 and was significantly lower compared to P19 and P60 and the two later timepoints analyzed (Figure 1F and Supplementary Tables 3, 4). Moreover, one-way ANOVA followed by a test for linear trend showed a significant increase in CB cell density form P19 to P120 in *Gli2*-cKO but not in control animals further indicating a changed course of CB interneuron maturation in the *Gli2*-cKO animals. Still, the density of CB interneurons stabilized in the *Gli2*-cKO mice after P60 and was then comparable to the one in controls (Figure 1F and Supplementary Tables 3, 4). These data suggest that in the absence of mesoprefrontal DA innervation, CB interneuron maturation is not properly regulated, and that the critical period for mesoprefrontal DA innervation to influence interneurons appears to be adolescence, typically defined between P23 and P60 in the mouse (Brust et al., 2015).

PV positive cells in the mPFC were detected across layers II-VI, with noticeably more PV expressing cells in the deeper layers than the upper layers (Figures 1H–J and Supplementary Figure 2). In the upper layers, the PV cell density did not show any significant changes across development and no changes between genotypes (Figure 1I and Supplementary Tables 3, 4). In the deeper layers, two-way ANOVA showed a significant effect of age (*F*_(6, 65)_ = 21.6, *p* < 0.0001) and *post-hoc* analysis revealed that the surge in cell density between P12 and P26 was significant in mice of both genotypes and significant differences between P12 and any of the later time points (P33-P120) were observed in control and *Gli2*-cKO mice (Figure 1J and Supplementary Tables 3, 4). Between P12 and P19 the increase in cell density was more pronounced in *Gli2*-cKO than in control mice and between P19 and P33 it was more pronounced in control than in *Gli2*-cKO mice. Notably, in the *Gli2*-cKO mice but not in controls, a significant decrease in cell density occurred between the peak plateau (reached at P26 and P33) and P60, but then stabilized to a level comparable to the control mice at subsequent time points. Accordingly, two-way ANOVA detected an effect of genotype (*F*_(1, 65)_ = 5.76, *p* = 0.0193) and the *post-hoc* test showed a significant difference between control and *Gli2*-cKO mice at P60 (Figure 1J and Supplementary Tables 3, 4). These data suggest that the maturation of PV interneurons is not properly regulated in the absence of mesoprefrontal DA innervation and that the critical period for the influence of DA innervation on PV interneuron maturation is adolescence, similar to our observations for CB interneurons.

### PV and *Gad1* expression levels are altered in PV interneurons in absence of mesoprefrontal dopaminergic innervation

**FIGURE 2:**
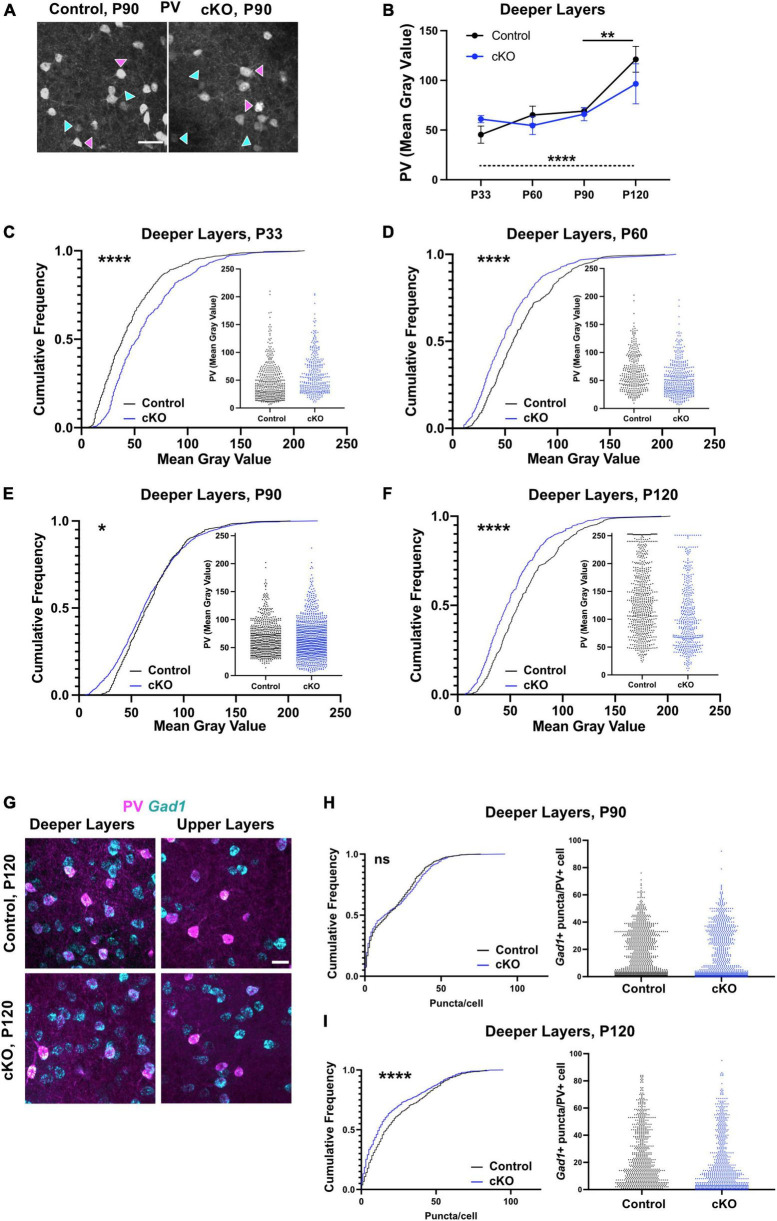
PV interneuron maturation is altered in absence of mesoprefrontal dopaminergic innervation in the mPFC. **(A–F)** Analysis of fluorescent intensity of mPFC immunostained for PV as a measure of PV expression level. **(A)** Representative images of immunofluorescent staining for PV in deeper layers of the P90 mPFC (coronal sections; scale bar: 50 μm). Magenta arrowheads indicate interneurons with strong signal for PV, cyan arrowheads interneurons with low signal for PV. **(B–F)** Quantification of intensity of immunofluorescent signal for PV in deeper layers of the mPFC from P33 to P120. *n* = 3–9 mice per group, *n* = 309–944 cells per group. **(B)** Data points represent the average intensity of PV fluorescence per group. Two-way ANOVA with age and genotype as the two main factors followed by Šídák’s multiple comparisons *post-hoc* test and one-way ANOVA followed by a *post-hoc* test for linear trend. **(C–F)** Data are represented as cumulative frequency plots and as scatter blots (small insets). **(G–I)** Analysis of *Gad1* expression levels in PV+ interneurons using fluorescent RNA *in situ* hybridization (RNA-FISH). **(F)** Representative images of immunofluorescent staining for PV (magenta) and RNA-FISH for *Gad1* (cyan) in deeper layers of the P120 mPFC (coronal sections; scale bars: 25 μm). **(G, H)** Quantification of *Gad1* expression levels (number of puncta per cell) in deeper layers of the mPFC at P90 and P120. Data are represented as cumulative frequency plots (left panel) and as scatter blots (right panel). *n* = 3–5 mice per group, *n* = 228–876 cells per group. **(C–F,H,I)** Statistical significance was assessed with a Mann-Whitney test comparing the control and the cKO group at each stage. Details on animal numbers are reported in Supplementary Table 3; details on statistical tests in Supplementary Table 4. Error bars indicate mean +/- SEM. ns, not significant, *p < 0.05, **p < 0.01, ****p < 0.0001.

To assess if the observed changes in PV cell density during adolescence were accompanied by changes in PV expression levels, the fluorescence intensity of the PV signal was analyzed during adolescence and adulthood in the deeper layers (Figures 2A–F). Comparison of the average intensity (mean gray value, MGV) in control and *Gli2*-cKO mice demonstrated no significant effect of genotype and no genotype x age interaction (two-way ANOVA), but a significant effect of age (*F*_(3, 31)_ = 11.87, *p* < 0.0001). A *post-hoc* test showed a significant increase in MGV in control but not in *Gli2*-cKO mice between P90 and P120. One-way ANOVA followed by a test for linear trend showed a significant systematic increase in MGV from P33 to P120 in control but not in *Gli2*-cKO animals (Figure 2B). To assess possible changes across the entire cell population studied, the data were analyzed using cumulative frequency distribution. At P33, the MGV in *Gli2*-cKO mice was shifted toward higher values relative to control mice (Figure 2C and Supplementary Tables 3, 4). In contrast, at P60-P120, a shift toward lower MGVs was evident in *Gli2*-cKO mice compared to control mice (Figures 2D–F and Supplementary Tables 3, 4). Scatter plots of the intensity distribution showed that in *Gli2*-cKO mice, the PV interneuron population between P60 and P120 contained a higher proportion of low-expressing PV interneurons (below a MGV of 50) compared to the control group (Figures 2D–F and Supplementary Tables 3, 4).

In various neuropsychiatric disorders and their animal models, altered PV levels are accompanied by changed *Gad1* levels (Hashimoto et al., 2003; Behrens et al., 2007; Zhang et al., 2008; Lee et al., 2013; Mukherjee et al., 2019). Thus, we assessed *Gad1* expression by fluorescent RNA *in situ* hybridization (RNA-FISH) at P90 and P120 (Figures 2G–I and Supplementary Tables 3, 4). At P90 *Gad1* gene expression (measured as puncta/PV interneuron) was comparable in control and *Gli2*-cKO (Figure 2H) while at P120, the cumulative frequency distribution curve in *Gli2*-cKO was shifted toward lower *Gad1* expression compared to the control group (Figure 2I). These results imply an overall dysregulation of the PV interneuron phenotype that persists into adulthood.

### Dopamine receptor transcript expression in mPFC is not obviously altered in absence of mesoprefrontal dopaminergic innervation

**FIGURE 3:**
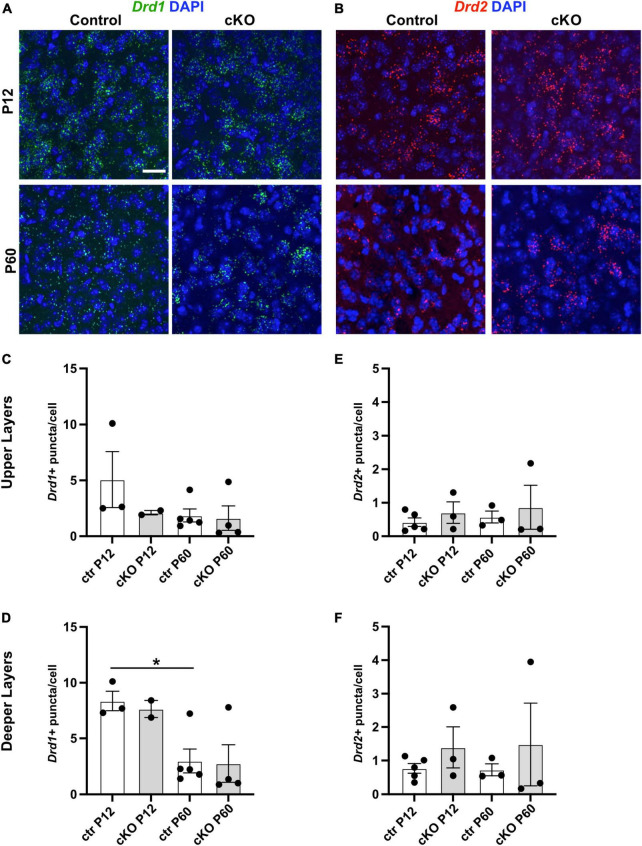
Dopamine receptor expression is not obviously changed in absence of mesoprefrontal dopaminergic innervation in the mPFC. **(A,B)** Representative images of fluorescent RNA *in situ* hybridization (RNA-FISH) for *Drd1* (**A**, green) or *Drd2* (**B**, red) and DAPI staining in deeper layers of the P12 and P60 mPFC (coronal sections; scale bar: 25 μm). **(C–F)** Quantification of *Drd1* **(C,D)** and *Drd2* **(E,F)** expression levels (number of puncta per cell) in upper and deeper layers of the mPFC at P90 and P120. Data points represent the average number of dots per cell per animal. Note that *Drd1* expression is higher in the deeper layers of the mPFC at P12 as compared to P60. *n* = 2–5 mice per group, *n* = 5730–11042 cells per group. Statistical significance was assessed with one-way Welch’s ANOVA followed by Dunnett’s T3 multiple comparisons *post-hoc* test. Details on animal numbers are reported in Supplementary Table 3; details on statistical tests in Supplementary Table 4. Error bars indicate mean +/- SEM. ns: not significant, *p < 0.05.

Loss of DA input to the striatum in adult life in Parkinson’s disease (PD) patients or in PD animal models leads to compensatory upregulation of dopamine receptor (DRD) expression (Hisahara and Shimohama, 2011). To determine if such a compensatory mechanism occurs in absence of mesoprefrontal DA innervation in the mPFC of *Gli2*-cKO mice, the expression of *Drd1* and *Drd2* transcripts was assessed by RNA-FISH in mPFC cells of control and *Gli2*-cKO mice. We focused on *Drd1* and *Drd2* since their expression is relatively well described in the mPFC (Islam et al., 2021). The analysis showed however no significant difference in expression between cells in the *Gli2*-cKO and control mPFC at P12 or P60 (Figures 3A–F and Supplementary Tables 3, 4). However, there was a significant decrease in *Drd1* expression in the deeper layers in the mPFC of control animals between P12 and P60, consistent with some previous reports (Figure 3D; Islam et al., 2021).

### Postnatal development of noradrenergic and serotonergic fibers in the mPFC of cKO and control mice

**FIGURE 4:**
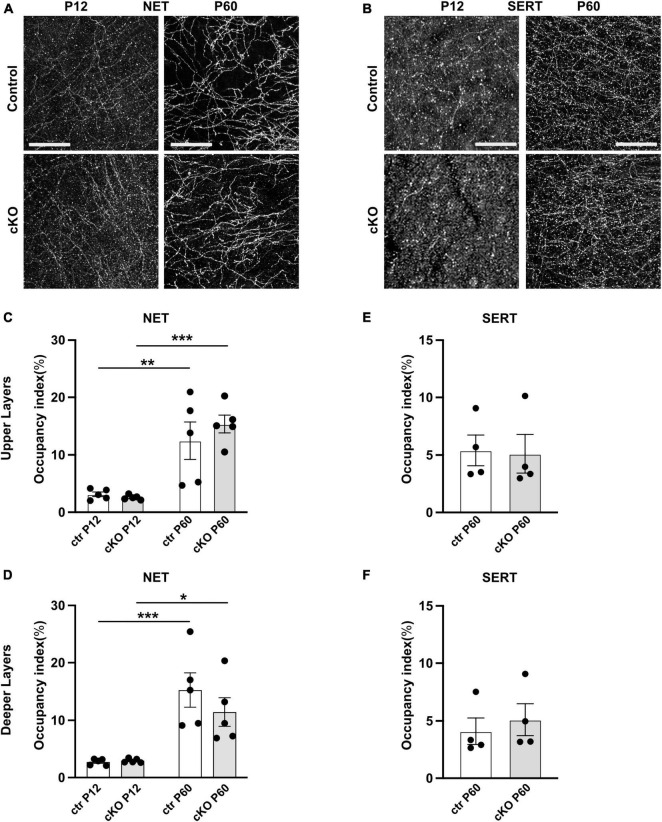
Noradrenergic and serotonergic innervation is not obviously changed in absence of mesoprefrontal dopaminergic innervation in the mPFC. **(A,B)** Representative images of immunofluorescent staining for NET **(A)** and for SERT **(B)** in the deeper layer of the mPFC at P12 and P60 (coronal sections; scale bars: 25 μm). **(C–F)** Quantification of the occupancy index of NET **(C,D)** and SERT **(E,F)** fibers in the upper and deeper layers of the mPFC. Data points represent the average occupancy index per animal. *n* = 5 mice per group. Statistical significance was assessed with two-way ANOVA with age and genotype as the two main factors followed by Šídák’s multiple comparisons *post-hoc* test for NET and unpaired *t*-test for SERT. Details on animal numbers are reported in Supplementary Table 3; details on statistical tests in Supplementary Table 4. Error bars indicate mean +/- SEM *p < 0.05, **p < 0.01, ***p < 0.001.

Since the monoaminergic systems in the mPFC interact and influence each other during development (Niederkofler et al., 2015; Garcia et al., 2019), we next investigated whether the absence of mesoprefrontal DA input elicits changes in the innervation pattern or density of serotonergic or noradrenergic projections. To this end, we performed immunostaining for norepinephrine transporter (NET) to label noradrenergic fibers and serotonergic transporter (SERT) to label serotonergic axons in the mPFC (Figures 4A, B). Since innervation density differs between upper and deeper mPFC layers for both noradrenergic and serotonergic fibers, analysis was separately performed for the two layers (Bang et al., 2012; Weele et al., 2018). At P12, in upper and deeper layers of the mPFC, the NET-immunoreactive fibers appeared thin, with intermittent varicosities along the axon. At P60 the innervation pattern was denser than at P12, and the axons appeared to be smoother and more uniform (Figure 4A). However, no difference in innervation density could be detected between the control and *Gli2*-cKO mice at either stage. Two-way ANOVA reported no effect of genotype but a significant effect of age in both upper (*F*_(1, 16)_ = 37.05, *p* < 0.0001) and deeper (*F*_(1, 16)_ = 30.29, *p* < 0.0001) layers. *Post-hoc* analysis revealed that between P12 and P60, the increase in area occupied by noradrenergic fibers was significant in mice of both genotypes (Figures 4C, D and Supplementary Tables 3, 4), consistent with increasing density of innervation during postnatal development described by others (Levitt and Moore, 1979).

At P12, SERT-immunostained axons in the mPFC appeared as very thin, short segments that were difficult to differentiate from background. At P60, SERT expression was stronger, and the axons could be visualized more easily. The morphological features of the fibers did not differ between control and *Gli2*-cKO mice at either stage (Figure 4B). Because of the relative weak SERT signal at P12, innervation density could only be quantified at P60. No significant difference was detected between genotypes at this stage (Figures 4E, F and Supplementary Tables 3, 4). In conclusion, the density of noradrenergic or serotonergic fibers in the mPFC was not obviously affected in the absence of mesoprefrontal DA innervation.

## Discussion

It is well established that the mesoprefrontal system modulates the signal-to-noise ratio and the E/I balance in local mPFC networks. These effects occur partially by dopamine activating complex intracellular signaling cascades ultimately resulting in altered activity of interneurons (Domenico and Mapelli, 2023). In the mPFC, interneurons and the mesoprefrontal input undergo a protracted maturation process (Caballero et al., 2014b; Islam et al., 2021). DA inputs have been shown to influence maturation of medium spiny neurons in the striatum and pyramidal neurons in the mPFC (Tseng and O’Donnell, 2005; Manitt et al., 2013; Lieberman et al., 2018) and modulation of DA signaling during a critical period in adolescence can rescue interneuron deficits in schizophrenia mouse models (Mukherjee et al., 2019; Mastwal et al., 2023). Given the evidence that dopamine modulates mature mPFC interneuron function and maturation of other neuronal populations, we asked whether the mesoprefrontal input also influences mPFC interneuron maturation. Here, we show that the complete lack of mesoprefrontal innervation throughout mPFC development in *Gli2*-cKO mice leads to an altered maturation process of PV and CB interneurons in the mPFC. Focusing on PV interneurons, we show that this population is particularly vulnerable to the lack of mesoprefrontal input, as PV interneurons in *Gli2*-cKO mice exhibit an inability to maintain adequate PV or *Gad1* expression levels. Despite the lack of mesoprefrontal innervation throughout the embryonic and postnatal development of the *Gli2*-cKO mice, these deficits do not become apparent until adolescence, and some persist into adulthood. Thus, our data demonstrate that the presence of adequate mesoprefrontal innervation is particularly critical for the normal maturational trajectories of PV and CB interneurons during adolescence. How this affects the maturation of local mPFC circuitry and whether there are lasting consequences for these local networks and mPFC function will need to be addressed in future studies. However, previous work has shown that inhibition of PV upregulation or suppression of PV neuronal activity during adolescence results in ineffective inhibition of pyramidal neurons, network dysfunction, and behavioral deficits (Caballero et al., 2020; Canetta et al., 2022), suggesting that a long-term effect of the observed interneuron maturation phenotype on mPFC networks is likely in the *Gli2*-cKO mice.

Previous studies have shown a developmental upregulation of PV mRNA expression in the dorsolateral PFC of humans (Fung et al., 2010) and an increase in PV protein level, PV immunofluorescence, and PV cell density in rodent mPFC during adolescence (Caballero et al., 2014a; Du et al., 2018). It has been suggested that the boost in adolescent PV expression is associated with an increase in excitatory glutamatergic transmission onto fast-spiking PV neurons (Tseng and O’Donnell, 2005, 2007). In this context, it is important to consider that a subset of the mesoprefrontal DA inputs have the ability to co-release glutamate (Mingote et al., 2019). Thus, PV interneuron maturation could be influenced by DA and GLU release from mesoprefrontal fibers. Indeed, we have shown previously that stimulation of mesoprefrontal DA fibers results in excitation of fast-spinking interneurons and that this excitatory input is missing in the mPFC of *Gli2*-cKO mice (Kabanova et al., 2015). On the other hand, dopamine could act on interneurons either indirectly, by changing the strength of excitatory inputs (e.g., from pyramidal neurons) (Dong et al., 2004; Tseng and O’Donnell, 2005; Manitt et al., 2013) or directly through influencing interneuron activity or activating downstream signaling cascades. In striatal medium spiny neurons of the direct pathway (*Drd1*-expressing), loss of DA input impacts on phosphatidylinositol 4,5-biphosphate (PIP2) levels resulting in decreased inwardly rectifying potassium currents and increased excitability of the neurons (Lieberman et al., 2018). In acute slices of the adolescent mPFC, dopamine has been shown to depolarize fast-spiking interneurons by suppressing a ‘leak’ potassium current and inwardly rectifying potassium current (Gorelova et al., 2002). Whether such depolarizing effects of dopamine also contribute to the maturation of mPFC PV interneurons will be interesting to study in the future. Finally, mDA neurons also express a range of neuropeptides. For mDA neurons expressing the genes encoding the neuropeptides *Cck* (*cholecystokinin*) and *Grp (Gastrin-releasing peptide*) it has been shown that a small subset of them projects to the mPFC (Anderegg et al., 2015; Kramer et al., 2018; Poulin et al., 2018). Whether these and potentially other neuropeptides are actually released from mesoprefrontal fibers remains to be demonstrated but given that neuropeptide receptors are expressed in the mPFC (Casello et al., 2022; Zhong et al., 2022), altered neuropeptide levels may also contribute to the maturation phenotype of interneurons that we observe in the absence of mesoprefrontal innervation.

GABAergic interneurons gate long-range inputs and generate cortical oscillatory activity and neural synchrony that underlies emotional and cognitive behavior (Sohal et al., 2009; Uhlhaas and Singer, 2011; Le Magueresse and Monyer, 2013; Murray et al., 2015). Inhibiting PV upregulation or suppression of PV neuron activity during adolescence leads to ineffective inhibition of pyramidal neurons, network dysfunction and behavioral deficits such as impairments in extinction learning and extradimensional set-shifting behavior (Caballero et al., 2020; Canetta et al., 2022). Indeed, we have shown previously that *Gli2*-cKO mice show altered perseverance behavior in response to changes in task contingency in a 5-choice serial reaction time task, while motor function, motivation and impulsivity were not affected (Kabanova et al., 2015). This is consistent with findings showing that cognitive flexibility is dependent on intact PV interneuron activity (Cho et al., 2015; Murray et al., 2015). The results presented here suggest that a dysregulated interplay between mesoprefrontal input and interneurons, not only in the mature mPFC but especially in the adolescent period, may be an important contributor to network alterations in neuropsychiatric disorders and disease models.

Finally, we found no evidence for compensatory changes in *Drd1/2* transcript levels or in the innervation density of serotonergic or noradrenergic axons in the *Gli2*-cko mPFC. However, possible compensatory changes in serotonin or noradrenaline release would need to be investigated using fluorescent sensors (Kubitschke and Masseck, 2024). This would be particularly interesting for the noradrenergic system, since hippocampal noradrenergic axons can release dopamine (Duszkiewicz et al., 2018). Thus, noradrenergic fibers in the mPFC may also release dopamine, and increased dopamine release from these fibers could potentially compensate for the lack of mesoprefrontal DA input in *Gli2*-cko mPFC.

## Data availability statement

The original contributions presented in this study are included in the article/Supplementary material, further inquiries can be directed to the corresponding author.

## Ethics statement

The animal study was approved by the Landesamt für Natur, Umwelt und Verbraucherschutz Nordrhein-Westfalen. The study was conducted in accordance with the local legislation and institutional requirements.

## Author contributions

SB: Writing – review and editing, Writing – original draft, Visualization, Validation, Supervision, Resources, Project administration, Methodology, Funding acquisition, Data curation, Conceptualization. KI: Writing – review and editing, Visualization, Methodology, Investigation, Formal analysis, Data curation, Conceptualization.

## Footnotes

1
http://cellprofiler.org/examples/#Speckles

## Supplementary material

Supplementary Figure 1 Calbindin (CB) expression in upper and deeper layers of the mPFC in control and *Gli2* cKO mice at different postnatal stages. Representative images of immunofluorescence staining for calbindin in deeper and upper layers of mPFC from P12-P120 (coronal sections; scale bar: 50 μm). Representative images for P90 are shown in Figure 1B.

Supplementary Figure 2 Parvalbumin (PV) expression in upper and deeper layers of the mPFC in control and *Gli2* cKO mice at different postnatal stages. Representative images of immunofluorescence staining for parvalbumin in deeper and upper layers of mPFC from P12-P120 (coronal sections; scale bar: 50 μm). Representative images for P90 are shown in Figure 1B.
