# Supplementary material for: The impact of the mesoprefrontal dopaminergic system on the maturation of interneurons in the murine prefrontal cortex
Source: Front Neurosci. 2024 Jul 5;18:1403402. doi: 10.3389/fnins.2024.1403402 (PMC11257905; doi:10.3389/fnins.2024.1403402)
Supplement: Supplementary file 1 [file Table_1.DOCX]

**Supplementary Table 1. Primary and secondary antibodies and fluorophore conjugated streptavidin used for immunostaining; probes used for fluorescent RNA *in situ* hybridization.**

| **1^st^ Antibody** | **Species** | **Dilution** | **Cat. No** | **RRID Identifier** | **Company** |
| --- | --- | --- | --- | --- | --- |
| Anti-Parvalbumin | Rabbit | 1:1000 | PV27 | AB_2631173 | Swant |
| Anti-Calbindin | Rabbit | 1:2000 | CB38a | AB_10000340 | Swant |
| Anti-Calretinin | Goat | 1:2000 | CG1 | AB_10000342 | Swant |
| Anti-NET | Rabbit | 1:2000 | 260003 | AB_2619974 | Synaptic Systems |
| Anti-SERT | Rabbit | 1:5000 | 24330 | AB_572209 | Immunostar |
| **2^nd^ Antibody** | **Species** | **Dilution** | **Cat. No** | **RRID Identifier** | **Company** |
| Anti-Goat Biotin | Donkey | 1:500 | 706-065-147 | AB_2340397 | Jackson ImmunoResearch |
| Anti-Rabbit Biotin | Donkey | 1:500 | 711-065-152 | AB_2340593 | Jackson ImmunoResearch |
| Anti-Rabbit Alexa 488 | Donkey | 1:500 | A-21206 | AB_2535792 | Thermo Fischer Scientific |
| Anti-Rabbit Alexa 546 | Donkey | 1:500 | A10040 | AB_2534016 | Thermo Fischer Scientific |
| Anti-Rabbit Alexa 647 | Donkey | 1:500 | A-31573 | AB_2536183 | Thermo Fischer Scientific |
| **Streptavidin** |  | **Dilution** | **Cat. No** |  | **Company** |
| Cy3 Streptavidin |  | 1:1000 | 016-160-084 |  | Jackson  ImmunoResearch |
| Alexa 647 Streptavidin |  | 1:500 | S32357 |  | Thermo Fischer Scientific |
| **Probe** |  |  | **Cat. No** |  | **Company** |
| *Mm-Drd1* |  |  | 461901 |  | Advanced Cell Diagnostic |
| *Mm-Drd2* |  |  | 406501 |  | Advanced Cell Diagnostic |
| *Mm-Gad1* |  |  | 400951 |  | Advanced Cell Diagnostic |
