# Supplementary material for: The impact of the mesoprefrontal dopaminergic system on the maturation of interneurons in the murine prefrontal cortex
Source: Front Neurosci. 2024 Jul 5;18:1403402. doi: 10.3389/fnins.2024.1403402 (PMC11257905; doi:10.3389/fnins.2024.1403402)
Supplement: Supplementary file 2 [file Table_2.docx]

**Supplementary Table 2. Image acquisition and Image analysis of RNA-FISH data**

MPI: Mean pixel intensity

| **Imaged structures** | **Age** | **Microscope** | **Objective** | **Software** | **Comments** |
| --- | --- | --- | --- | --- | --- |
| PV, CR, CB, cell density | P19, P26, P33, P60 | Zeiss AxioObserver Z1 | EC PlnN 20x/0.5 | Zen blue software, Zeiss | Single plane |
| PV, CR, CB, cell density | P12 | Zeiss Axio Scan.Z1 slide scanner | PlanNeofluar 20x/0.5 | Zen 2 software, Zeiss | Single plane |
| PV, CR, CB, cell density | P90, P120 | Zeiss AxioObserver CSU-W1 confocal scanner unit | PlanApochromat 20x/0.8 | VisiView software, Visitron | Z-stack of 8 planes, z-step: 2 μm |
| PV, fluorescence intensity | P33, P60, P90, P120 | Zeiss AxioObserver CSU-W1 confocal scanner unit | PlanApochromat 20x/0.8 | VisiView software, Visitron | Z-stack of 8 planes, z-step: 2 μm |
| Noradrenergic and serotonergic innervation | P12, P60 | Leica TCS SP8 upright microscope | HC PL APO 63X/1.3 Gly | LAS AF v3.x, Leica | Z-stack of 67 planes, z-step: 0.15 μm |
| *Drd1, Drd2,* RNA-FISH signal | P12, P60 | Zeiss AxioObserver CSU-W1 confocal scanner unit | C-Apochromat, 40x/1.2 water | VisiView software, Visitron | Z-stack of 12 planes, z-step: 1 μm |
| *Gad1,* RNA-FISH signal | P90, P120 | Zeiss AxioObserver CSU-W1 confocal scanner unit | C-Apochromat, 40x/1.2 water | VisiView software, Visitron | Z-stack of 12 planes, z-step: 1 μm |
| **Probes** | **Threshold** | **Size of puncta** | **Area in which puncta are analyzed** |  |  |
| *Gad1* | 2 standard deviations above MPI | 3-20 pixels | Cell soma marked by PV expression |  |  |
| *Drd1, Drd2* | 3 standard deviations above MPI | 3-20 pixels | In DAPI+ nuclei and within 20 pixels of the DAPI+ nuclei (to include cytoplasm) |  |  |
