# Supplementary figures and images for: The impact of the mesoprefrontal dopaminergic system on the maturation of interneurons in the murine prefrontal cortex
Source: Front Neurosci. 2024 Jul 5;18:1403402. doi: 10.3389/fnins.2024.1403402 (PMC11257905; doi:10.3389/fnins.2024.1403402)

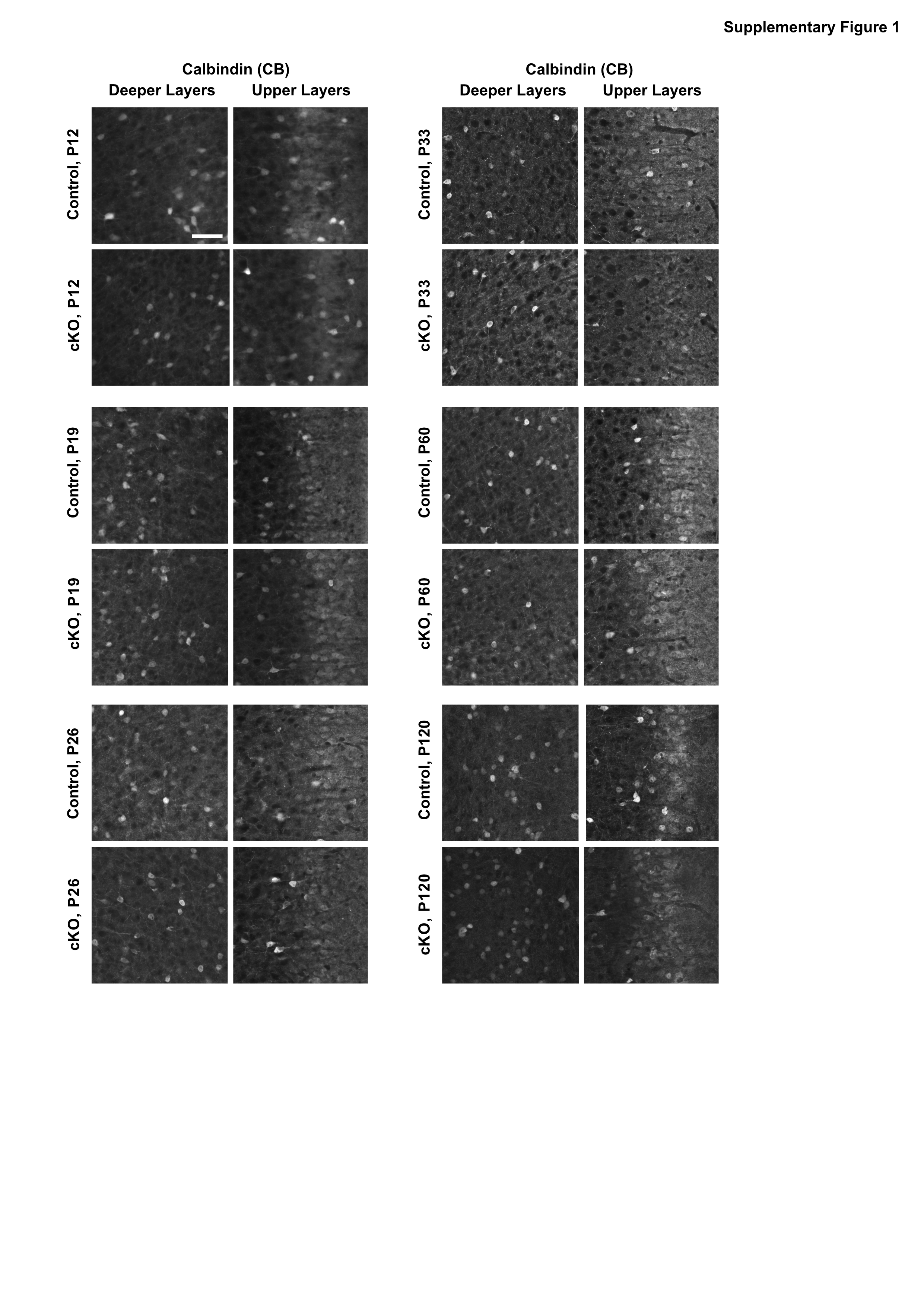

Supplement: Supplementary Figure 1 — Calbindin (CB) expression in upper and deeper layers of the mPFC in control and Gli2 cKO mice at different postnatal stages. Representative images of immunofluorescence staining for calbindin in deeper and upper layers of mPFC from P12-P120 (coronal sections; scale bar: 50 μm). Representative images for P90 are shown in Figure 1B. [file Image_1.TIFF]

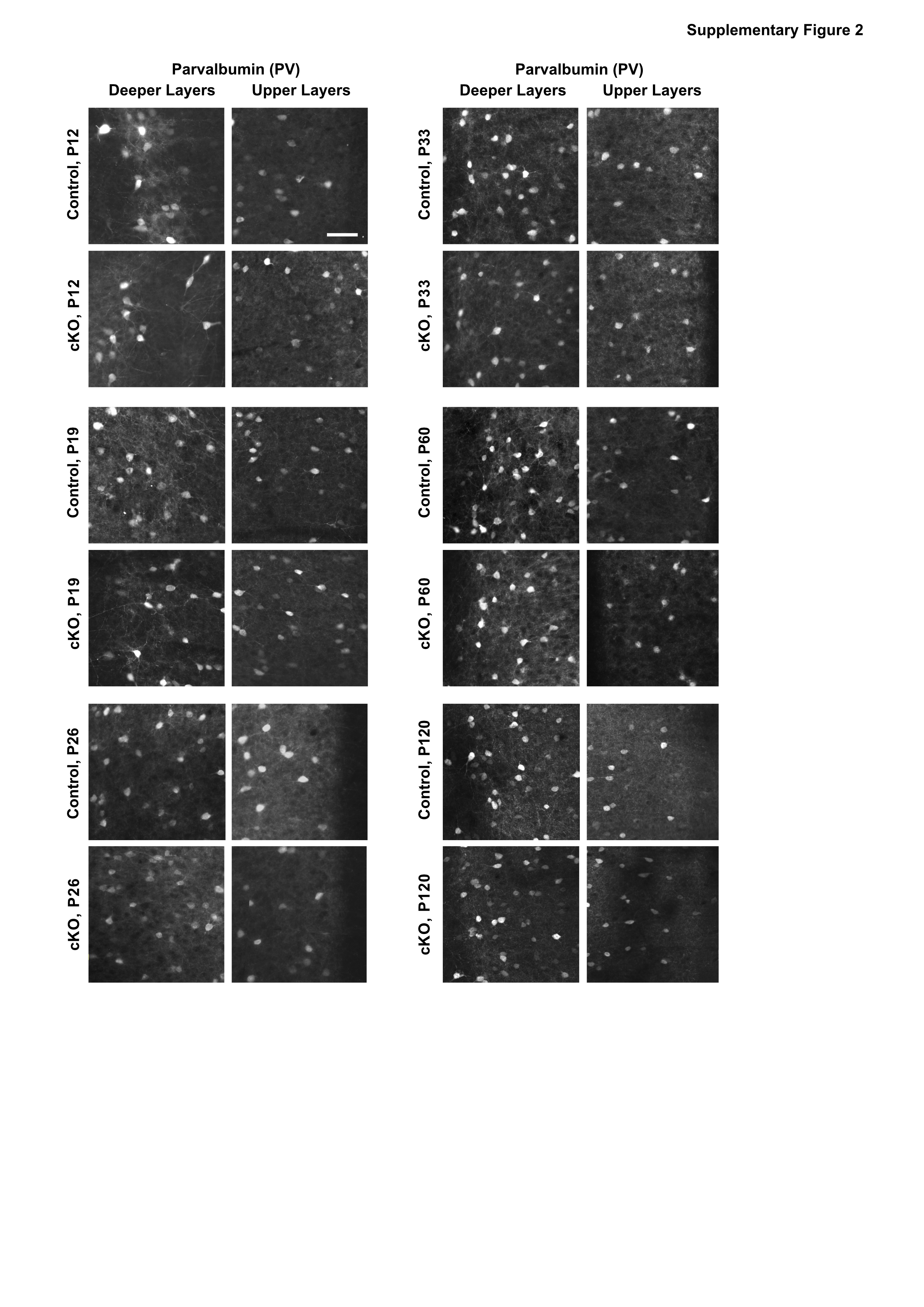

Supplement: Supplementary Figure 2 — Parvalbumin (PV) expression in upper and deeper layers of the mPFC in control and Gli2 cKO mice at different postnatal stages. Representative images of immunofluorescence staining for parvalbumin in deeper and upper layers of mPFC from P12-P120 (coronal sections; scale bar: 50 μm). Representative images for P90 are shown in Figure 1B. [file Image_2.TIFF]
